# Analysis of Massive Online Medical Consultation Service Data to Understand Physicians’ Economic Return: Observational Data Mining Study

**DOI:** 10.2196/16765

**Published:** 2020-02-18

**Authors:** Jinglu Jiang, Ann-Frances Cameron, Ming Yang

**Affiliations:** 1 Binghamton University Binghamton, NY United States; 2 HEC Montreal Montreal, QC Canada; 3 Central University of Finance and Economics Beijing China

**Keywords:** Web-based health services, remote consultation, machine learning, data mining, decision tree, patient involvement

## Abstract

**Background:**

Online health care consultation has become increasingly popular and is considered a potential solution to health care resource shortages and inefficient resource distribution. However, many online medical consultation platforms are struggling to attract and retain patients who are willing to pay, and health care providers on the platform have the additional challenge of standing out in a crowd of physicians who can provide comparable services.

**Objective:**

This study used machine learning (ML) approaches to mine massive service data to (1) identify the important features that are associated with patient payment, as opposed to free trial–only appointments; (2) explore the relative importance of these features; and (3) understand how these features interact, linearly or nonlinearly, in relation to payment.

**Methods:**

The dataset is from the largest China-based online medical consultation platform, which covers 1,582,564 consultation records between patient-physician pairs from 2009 to 2018. ML techniques (ie, hyperparameter tuning, model training, and validation) were applied with four classifiers—logistic regression, decision tree (DT), random forest, and gradient boost—to identify the most important features and their relative importance for predicting paid vs free-only appointments.

**Results:**

After applying the ML feature selection procedures, we identified 11 key features on the platform, which are potentially useful to predict payment. For the binary ML classification task (paid vs free services), the 11 features as a whole system achieved very good prediction performance across all four classifiers. DT analysis further identified five distinct subgroups of patients delineated by five top-ranked features: previous offline connection, total dialog, physician response rate, patient privacy concern, and social return. These subgroups interact with the physician differently, resulting in different payment outcomes.

**Conclusions:**

The results show that, compared with features related to physician reputation, service-related features, such as service delivery quality (eg, consultation dialog intensity and physician response rate), patient source (eg, online vs offline returning patients), and patient involvement (eg, provide social returns and reveal previous treatment), appear to contribute more to the patient’s payment decision. Promoting multiple timely responses in patient-provider interactions is essential to encourage payment.

## Introduction

### Background

Online health care solutions are increasingly popular [1-3], with reports that they are preferred by more than 70% of patients [4]. This study focuses on *multisided online medical consultation platforms* where various health care providers from different hospitals and medical institutes provide remote medical consultation services to patients. This type of digital health care service is experiencing significant growth and research attention [5]. These platforms offer many benefits, such as reduced medical costs, improved medical service efficiency, more efficient health care resource distribution, and fewer health care resource shortages in remote areas [2,6-9].

Despite the popularity and potential benefits, some online medical consultation platforms are struggling to attract and retain patients who are willing to pay for these services, for example, patient dissatisfaction after an initial failed experience, fear that diagnoses are made with limited consideration of patients’ medical history, and concerns about privacy may impede patients’ use of online consultation [10,11]. In addition, online medical consultation usually follows the Pareto principle in that 80% of the services are provided by 20% of the physicians on the platform [1], suggesting that many health care service providers on the platform have the challenge of attracting patients and standing out in the crowd of physicians who can provide comparable services [6,12]. To entice patients to their platform and promote payment, many platforms employ a multitiered pricing strategy that allows the coexistence of free (ie, the free trials) and paid versions (ie, the premium) of services [13]. As a consequence, patients may be more willing to pay for the service, and physicians may be able to access a broader range of patients.

Several features associated with patient payment in online medication consultation platforms have been frequently examined by previous research. Physician reputation—both online and offline—is the most frequently examined physician characteristic [14]. As medical consultation is highly professional, physicians need to be credible or trustworthy to attract and retain paying patients. A physician’s affiliation, seniority, and location are usually used as proxies for reputation [8,9,15,16]. Patient evaluation, which is the feedback left on the platform by previous patients about the physician, is also frequently examined [2,6,17]. It is often displayed in the form of ratings, stars, reviews, and virtual gifts. This feedback is visible to other patients on the platform and may serve as signals of service quality, which impact patients’ willingness to pay. Although less frequently examined, patient-physician interaction may be an important feature as well. The frequency and depth of interaction on the platform (eg, the amount of service or the frequency of service) show the ability and willingness of a physician to provide high-quality service, which may influence patient payment [3,15,18,19].

### Gaps and Objectives

This existing research is useful; however, these service and physician-related features are often examined in isolation and often using a linear regression approach. Thus, the understanding of how various features interact to generate impacts is currently lacking—although some features might be important enough to generate impacts on their own, others may only have impacts when combined with other features. To extend existing research, new approaches are needed, which take advantage of the massive data on these platforms and help uncover the complex dynamics between these various features and their interactions and payment. Thus, the objectives of this study were to determine (1) the important features of online medical consultation services that are associated with patient payment, as opposed to free trial–only appointments; (2) the relative importance of these features; and (3) how these features interact, linearly or nonlinearly, in relation with payment. We focus on mining feature importance because knowing the features (and their interactions), which influence payment, will help platforms and physicians identify high-value online medical consultations. Although many features may impact payment, we are particularly interested in those, which are publicly visible on the platform, such as characteristics of physicians and their interaction with patients and patient feedback, rather than nonvisible features, such as patients’ economic status and their general attitude toward technology. This is because publicly visible features contain information and signals that, through observational learning and social influence [20-23], may influence patient payment.

To this end, we examine a massive dataset from the largest China-based online medical consultation platform (1.5 million patient-physician consultation records) spanning 10 years. Predictive models are developed by employing classic machine learning (ML) procedures (ie, feature selection, hyperparameter tuning, model training, and validation) with logistic regression (LR), simple decision tree (DT), random forest (RF), and gradient boost (GB) classifiers. The importance ranking of these features is identified through regression coefficients, level of DT splits, and feature importance scores provided by RF and GB algorithms.

## Methods

### Empirical Setting and Dataset

Our empirical setting is a multisided online medical consultation platform based in China. It is one of the largest medical platforms, and more than half a million physicians from over 9400 hospitals have set up their profiles and provided consultation services on the platform. The platform follows a service model that allows the coexistence of free and paid consultation services (see Multimedia Appendix 1 for more details).

Our dataset includes 10 years of consultation records (approximately 2.3 million records from January 2009 to August 2018) between patient-physician pairs from three departments that have received the most visits (ie, pediatrics, gynecology, and dermatology, according to the platform report) across six geographic areas—three of the areas are those with the richest health care resources (Beijing municipality, Guangdong province, and Zhejiang province) and three are remote areas with the fewest health care resources (Shanxi province, Tibet province, and Qinghai province). Each record is a consultation history that includes picture- and text-based dialogs and service purchase records between patient *i* and physician *j* (see Multimedia Appendix 1).

### Machine Learning Task and Initial Feature Selection

Our focal outcomes are whether a consultation record includes payment and the relative importance of the features on the platform that can predict payment. Although a consultation record may include multiple times of payments, we do not consider payment intensity or types. Accordingly, the objective of our ML task is to solve a binary classification problem—classifying consultation records into free services only (labeled as *free*) or those including some type of financial payment (labeled as *paid*). The consultation with a *paid* label is our positive class in ML prediction.

The initial 18 features were identified by drawing on variables that have been examined in previous studies (see Table 1 for definition and coding of features) and were consistently visible on the platform. Features that are visible to platform users (eg, visitors, patients, and physicians) may influence payment, as they potentially allow patient learning and valuation to occur before the actual consumption of the consultation service. Although the importance of online physician reputation has been demonstrated in previous studies [19], physicians’ online rating was not included in this study. Owing to the changes in platform design, online reputation scores (eg, stars, ratings, and reviews) are not consistent over time. In addition, we observed that most physicians have very good ratings with little variation (mean 3.80, SD 0.34), which would have made this feature less useful as a predictor. This ceiling effect has been reported in the previous study using the same context [1,9]. However, features such as social returns and service intensity were included and can reflect physicians’ online reputation to some extent [9].

**Table 1:** Key predictive features and coding description.

Feature		Description	Reference
**Physician reputation related**			
	Hospital ranking^a^	[Ranking 1] Equals 1 if primary care hospital, 0 otherwise. [Ranking 2] Equals 1 if secondary care hospital, 0 otherwise.	[2,3,9,14,19]
	Physician seniority	[Title 1] Equals 1 if chief physician, 0 otherwise. [Title 2] Equals 1 if associate chief physician, 0 otherwise.	[2,3,9,14,19]
	Hospital location	[Loc] Equals 1 if health care resource–rich areas, 0 otherwise.	[9,15]
	Physician tenure	[Tenure] The number of months the physician has been registered on the platform.	[15,19]
	Service intensity	[Intensity] The average number of patients served per month during the physician’s tenure (=total patients served/tenure).	[7]
**Patient related**			
	Previous formal examination	A function provided by the platform allowing patients to reveal their medical status: Status 1: no formal health care examination before the consultation. Status 2: a formal health care examination before the consultation. Status 3: private (ie, detailed consultation information is not directly visible by other patients). (coded into dummies) [PriorExam] Equals 1 if none, 0 otherwise. [Private] Equals 1 if set as private, 0 otherwise.	N/A^b^
	Offline connection	[Offline] A check-in function provided by the platform to indicate patients’ offline connection with the physicians. Equals 1 if the patient used the check-in function, 0 otherwise.	[16]
**Service delivery related**			
	Service duration	[Duration] Number of days between the initial post and last post of patient i’s interaction with physician j.	N/A
	Total dialog	[TotalD] Total number of posts within patient i’s interaction with physician j.	[18]
	Physician posts	[PhysicianP] Number of posts initiated by physician j within patient i’s interaction with physician j.	[3]
	Response rate	[Response] The rate of response of a physician (=PhysicianP/TotalP).	N/A
	Answer frequency	[Answer_frq] The average number of answers (including notifications and reminders) by the physician per day during patient i’s interaction with physician j (=PhysicianP/Duration).	N/A
	Social return	[Social] A function provided by the platform to allow patients to send virtual gifts to the physician. Equals 1 if patient i gave any virtual gift to physician j at any time during patient i’s interaction with physician j.	[2,3,12,15,18,19]
**Patient involvement related**			
	Patient posts	[PatientP] Number of posts initiated by patient i within that patient’s interaction with physician j.	[3]
	Question frequency	[Question_frq] The average number of posts by the patient per day during patient i’s interaction with physician j (=PatientP/Duration).	N/A

^a^Hospital ranking in China is a three-tier system (primary, secondary, and tertiary institutions) based on the hospital’s ability to provide medical care, education, and research; thus, physicians who have been able to secure a position at a primary care hospital are generally considered to be of higher reputation [24].

^b^Not applicable.

### Data Cleaning and Analysis Pipeline

First, data were prepared by removing consultation records that did not fit the scope of the study (eg, consultation occurred before 2009 and after 2018 and samples with unqualified tags). We also excluded records with over 50% of missing values (N=84,582) and outliers using the 95% quantile as the threshold (N=674,767; see Multimedia Appendix 2 for a detailed description of data cleaning procedure).

In the second step, four data-driven feature selection techniques were applied to identify the right features to use in the ML classification (low variance filtering, high correlation filtering, backward feature selection, and forward feature selection) [25,26]. The objective of this procedure is to find the features that are highly correlated with the outcome but ideally uncorrelated with each other [27] so that the resulting features can build a relatively parsimonious model (see Multimedia Appendix 3 for a detailed description of feature selection procedure).

**Figure 1:**
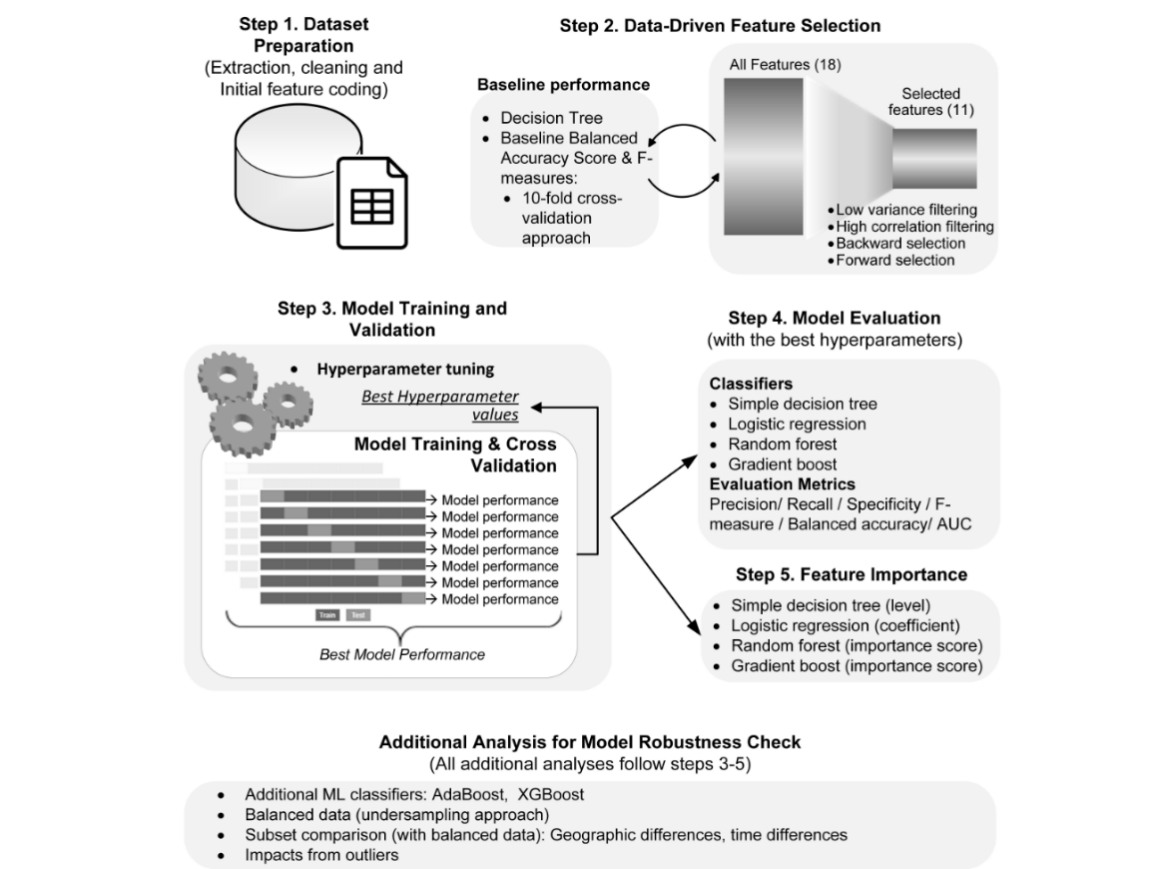
Analysis pipeline. AUC: area under the receiver operating characteristic curve; ML: machine learning.

In step 3, the ML model was constructed through three nested procedures: hyperparameter optimization, model training, and validation (see Figure 1). Four common ML classifiers were purposefully chosen—LR, DT, RF, and GB—because they are mainstream ML techniques for classification problems [13] accessible by general data consumers through data analysis tools and platforms (eg, Python, R, SAS, and RapidMiner). LR was used in previous studies with small datasets [2], and the latter three are tree-based approaches with different resampling strategies and cost function optimization techniques (ie, boosting vs bagging and gradient descent algorithm). Depending on the ML classifier, different sets of hyperparameters need to be configured to ensure that the algorithm reaches its best classification performance (see Multimedia Appendix 2 for a detailed explanation of optimization and analysis procedures). We conducted our analysis on the KoNstanz Information MinEr platform.

The performances of the resulting ML models were compared in step 4. We used six evaluation metrics, which are commonly accepted in ML classification and can reflect different aspects of ML model performance (eg, correctly assign the paid services with a paid label vs the probability that an ML classifier will successfully classify a case in the right class) [28,29] (see Multimedia Appendix 2 for detailed explanation of our evaluation metrics).

We investigated research objectives 2 and 3 through step 5, which examines feature importance. The four classifiers that we used provide different feature importance indicators—the regression coefficients in LR, level of splits for DT, and feature importance indices for both GB and RF (see Multimedia Appendix 2, and the study by Friedman [30]).

For steps 3 to 5, there are some particularities of our data that may bias our results (eg, imbalanced data). Thus, we perform several additional tests to examine the robustness of the model. The results of these additional analyses indicate that our model is robust to sample distribution (eg, imbalances, classes, and outliers) and potential systematic differences (eg, geographic location and market changes), as indicated by only minor changes in the model performance measures (see Multimedia Appendix 4 for the results of these additional analyses).

## Results

### Feature Selection Results and Descriptive Statistics

After data cleaning, 1,582,564 qualified records remained for further analysis. Among these records, 1,089,662 (68.85%) were free trial–only, whereas 492,902 (31.15%) involved at least one premium payment. After performing four feature selection techniques (step 2, see Multimedia Appendix 3), we retained the ones that are selected by forward, backward, low variance filtering, and high correlation filtering approaches. In response to our first research objective regarding which features of online medical consultation services are associated with patient payment, our feature selection analysis suggested 11 key features (see Table 2)—the seven eliminated features were thus considered as less useful because of either high correlation with the included features (ie, redundant features) or low variance explained (ie, low explanatory power).

**Table 2:** Summary statistics of features.

Service feature		All, mean (SD)	Free-only^a^, mean (SD)	Paid^a^, mean (SD)	All (minimum, maximum)	Free-only (minimum, maximum)	Paid (minimum, maximum)
**Physician reputation related**							
	Hospital ranking 2	0.02 (0.15)	0.02 (0.16)	0.01 (0.12)	0, 1	0, 1	0, 1
	Physician title 1	0.46 (0.5)	0.43 (0.5)	0.53 (0.5)	0, 1	0, 1	0, 1
**Patient related**							
	PriorExam	0.19 (0.39)	0.06 (0.24)	0.47 (0.5)	0, 1	0, 1	0, 1
	Private	0.08 (0.27)	0.07 (0.25)	0.1 (0.3)	0, 1	0, 1	0, 1
	Offline connection	0.71 (0.45)	0.87 (0.33)	0.36 (0.48)	0, 1	0, 1	0, 1
**Service delivery related**							
	Total dialog	7.44 (6.38)	6.27 (5.03)	10.04 (8.06)	1, 35	1, 31	1, 35
	Response rate	0.19 (0.16)	0.18 (0.16)	0.2 (0.17)	0, 0.875	0, 0.75	0, 0.875
	Answer frequency	0.22 (0.33)	0.24 (0.35)	0.18 (0.29)	0, 1.25	0, 1	0, 1.25
	Social return	0.18 (0.38)	0.18 (0.38)	0.18 (0.29)	0, 1	0, 1	0, 1
**Patient involvement related**							
	Patient posts	5.79 (5.10)	5.05 (4.38)	7.43 (6.10)	1, 28	1, 28	1, 28
	Question frequency	1.11 (1.2)	1.2 (1.24)	0.92 (1.07)	0, 5.5	0, 5.5	0, 5.5

^a^Mean differences between free and paid services are all significant (*P*<.001), except for social return (*P*=.025).

### Machine Learning Model Performance and Feature Importance Ranking

Next, the overall model performance was examined (step 4; see Table 3). As we have an imbalanced dataset (ie, the ratio between paid and free-only services is around 1:2), area under the receiver operating characteristic curve (AUC), F measure, and balanced accuracy are less biased and more informative than other measures. GB exhibited the best overall performance (balanced accuracy=0.973, F measure=0.97, and AUC=1). However, all classifiers performed well, indicating that our predictive model with 11 selected features exhibits significant classification performance. Explanation of each measure is presented in Multimedia Appendix 2.

In investigating our research objective on the relative importance of the 11 features, the four ML classifiers yielded relatively consistent results in the top-ranked and low-ranked features, whereas the ones in the middle were less consistent (Table 4). Offline connection, response rate, social return, total dialog, diagnoses from a prior examination, and private status consistently ranked high, whereas physician title, question frequency, and the second-tier hospital ranking were consistently ranked low.

**Table 3:** Machine learning model performance evaluation.

Model performance measurement	Logistic regression	Decision tree	Gradient boost	Random forest
Recall	0.851	0.949	0.952	0.908
Precision	0.896	0.989	0.988	0.984
Specificity	0.956	0.995	0.995	0.993
F measure	0.873	0.969	0.970	0.944
Balanced accuracy	0.903	0.972	0.973	0.951
Area under the receiver operating characteristic curve	1.000	0.988	1.000	0.988

**Table 4:** Key features listed in descending order of importance.

Service feature	Logistic regression (coefficient^a^)	Decision tree (level of splits)	Gradient boost (importance, %)	Random forest (importance, %)
1	Response rate (−13.89)	Offline connection (1)	Offline connection (30)	Offline connection (24)
2	Offline connection (−4.99)	Social return (2)	Total dialog (30)	PriorExam (20)
3	Social return (−3.11)	Total dialog (2)	Response rate (25)	Total dialog (18)
4	Patient posts (−2.63)	Private (3)	Social return (8)	Response rate (17)
5	Total dialog (2.47)	Response rate (3)	Private (6)	Patient post (9)
6	PriorExam (1.70)	PriorExam (4)	Patient posts (1)	Social return (7)
7	Private (−0.99)	Answer_frq (4)	PriorExam (0)	Private (2)
8	Ranking 2 (−0.305)	Patient posts (6)	Answer_frq (0)	Answer_frq (2)
9	Answer_frq (−0.14)	Question_frq (6)	Question_frq (0)	Question_frq (1)
10	Question_frq (−0.13)	Ranking 2 (8)	Title1 (0)	Title1 (0)
11	Title1 (−0.089)	Title 1 (9)	Ranking 2 (0)	Ranking 2 (0)

^a^For logistic regression, a regularization procedure (see Multimedia Appendix 2) is applied, so large weight coefficients are penalized for avoiding overfitting. All coefficients are significant (*P*<.001).

### Interpreting Key Patient Subcategories Based on Feature Configurations

**Figure 2:**
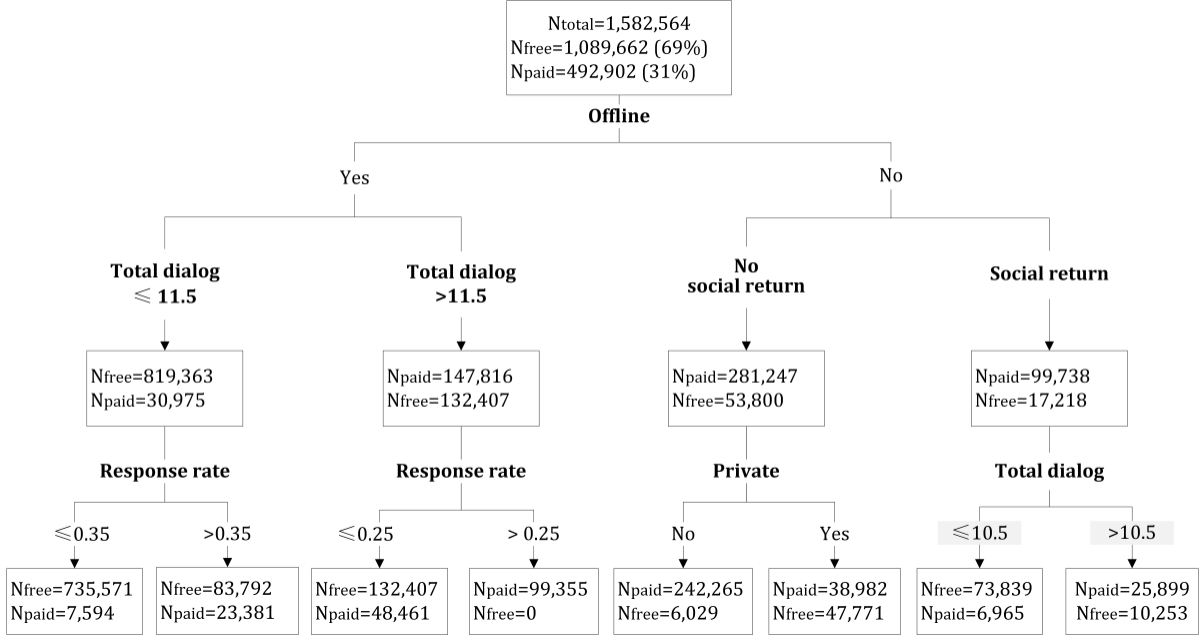
Decision tree for identifying patient subgroups with the full dataset.

To address the third research objective, we examined how these features interact in relation to patient payments. A tree structure was used because it explicitly displays the feature hierarchies and classification outcomes at each tree split. Five key feature configurations emerged, which describe five subgroups of patients who interact with physicians differently, yielding different payment outcomes. By applying the learned tree structure on the full dataset, these five subgroups covered 85.2% of the total population, using a combination of only four key features (ie, offline, total dialog, response rate, and social return). Note that the DT algorithm has the capability to fully classify the whole population (in our case, at 10 layers), but the configurations become complex and practically less useful. Thus, we used the subgroups up to the third layer (see Figure 2 and Table 5).

**Table 5:** Decision tree–based configuration of feature contributions.

Top feature configurations^a^		Number of cases in the node, n	Dominant outcome	Percentage of dominant cases, n (%)
**Subgroup 1: These configurations suggest a simple type of follow-up service resulting from previous offline diagnoses**				
	Offline AND low total dialog (≤11.5)	850,338	Free	819,363 (96.36)
	Offline AND low total dialog (≤11.5) AND low response rate (≤0.35)	743,165	Free	735,571 (98.98)
**Subgroup 2: This configuration suggests a complex service extension from the previous offline diagnoses, which requires intensive patient-provider interaction.**				
	Offline AND high total dialog (≤11.5) AND high response rate (>0.25)	99,355	Paid	99,355 (100)
**Subgroup 3: These configurations suggest the patient has no offline connection with the physician but is paying a premium for the online consultation rather than using a social return to show gratitude.**				
	Nonoffline AND no social return	335,047	Paid	281,247 (83.94)
	Nonoffline AND no social return AND nonprivate	248,294	Paid	242,265 (97.57)
**Subgroup 4: This configuration suggests the patient has no offline connection with the physician, has a less intensive online consultation experience, and offers a social return as compensation instead of payment.**				
	Nonoffline AND social return AND low total dialog (≤10.5)	80,804	Free	73,839 (91.38)
**Subgroup 5: This configuration suggests the patient has no offline connection with the physician and engages in an intensive online interaction, providing both payment and a social return as compensation.**				
	Nonoffline AND social return AND high total dialog (>10.5)	36,152	Paid	25,899 (71.64)

^a^For each tree split, if no dominant outcome emerges (ie, free cases <80% or paid cases <70% at the focal split), we do not consider it as an important subgroup because additional service features are required to better classify these cases.

We can observe that patients who have previous offline consultations with the physician are less likely to pay. It is possible that these patients tend to take free opportunities to clarify simple unsolved issues after their offline visits, as indicated by increasing the proportion of free services in the presence of low total dialog and low response rates from the physicians (subgroup 1). However, if complex issues emerge, these patients may still prefer to return to the offline health care channel rather than pay for the premium online service.

A second type of returning patients (subgroup 2) may have complex issues and decide to stay online and pay. This represents a complex service extension: these returning patients may have complex issues that require highly interactive patient-physician communication. Thus, these returning patients frequently communicate with the physicians (probably because of the complexity of the issue) and receive frequent responses, which, in turn, are associated with a high probability of payment.

For online patients who have no prior connection with the physician, those who do not provide social returns (eg, thank you letters and virtual gifts) seem more likely to pay (subgroup 3). There may be a psychological compensation effect [31] where giving virtual gifts substitutes for the actual payment and balances the sense of *guilt* after receiving free services. However, in cases where the service between patients and physicians with no offline connection is highly interactive (ie, large amount of dialog), patients provide both virtual gifts and premium payment to show their appreciation (subgroup 4 vs subgroup 5).

The high-level presence of *private* in one of the tree branches deserves more attention. *Privacy* represents a function provided by the platform, which allows patients to set their dialogs as private, so they cannot be viewed by other people. From previous studies, we know that one of the major reasons that patients do not use online health care services is privacy concerns [32,33]. Patients who use this function may have a higher privacy concern than those who do not use it. As online medical consultation requires patients to reveal sensitive health-related information, patients who allow this information to be publicly displayed probably have lower privacy concerns and may be more likely to be more engaged in the online consultation and subsequent diagnosis. Owing to this heightened engagement, they may be more likely to pay after the initial free interactions (subgroup 3).

In summary, the source of patients (offline returning or online directly) seems to be a key differentiator for payment, which may be because of the different motivations and service requirements inherent in these two types of patients. Patient-physician interaction representing service delivery quality is another key differentiator (eg, total dialogs, response rate, and patient posts), which also indicates the importance of patient involvement and physician’s timely response during the consultation. Privacy setting and social return, two features pertaining to the platform functionality, play important roles as well.

## Discussion

### Principal Findings

In this study, we focused on online medical consultation, a type of emerging digital health care service that has received much attention in recent years. Our objective was to understand the features of online medical consultation services that contribute to payment so that the platform can identify high-value services and take actions to better manage service providers and their offerings. As an initial study using ML approaches to identify key features and to make predictions, we did not aim to incrementally improve prediction accuracy by engineering the features or developing new algorithms. Rather, our goal was to develop a predictive model that has both sufficient explanatory power and practical interpretability so that it can be used by medical consultation platforms and service providers.

The high performance across the ML algorithms demonstrates that our 11-feature model is a useful predictive tool (research objective 1). In terms of feature importance (research objectives 2 and 3), our results show that although physician reputation is important, service delivery quality and patient involvement appear to contribute more to the payment. We further identified five patient subgroups based on DT feature configurations. The configurations show how features related to patient characteristics, platform functionalities, and patient-provider interaction are combined to result in different payment outcomes. These configurations highlight the offline connection and responsive service delivery as key differentiators for payment vs free trial–only services.

### Limitations

First, decisions made during the feature selection procedure may cause bias in the subsequent analysis. Although the results of this study achieved satisfactory overall performance, a different set of features that are comparable with the current ones can be used to cross-validate our model.

Second, although the platform provides various long- and short-term service options, to ensure consistency in data cleaning and interpretability of results, we only included short-term services based on the service tags available. However, future research should examine long-term service subscription, as patients’ decision-making criteria can be very different than for short-term service subscription.

Third, considering problems with data quality and limited variability, we did not include the platform’s online physician reputation ratings. However, future research could focus on physicians whose ratings do vary over time to observe how noticeable changes in ratings influence payment.

Fourth, our analysis was based on the Chinese context. Considering the cultural differences and health care regulations, our results may have limited generalizability to other contexts. However, the mechanisms and types of interactions that have been found are generic enough to be promoted and managed in different online medical consultation platforms and in different countries. Furthermore, the Chinese context itself is quite large and should be of interest on its own.

### Comparison With Prior Work

Although the majority of features in our predictive model were examined in existing research on payment for online medication consultation, several new features specific to this type of platform and some surprising differences from existing research also emerged. Unexpectedly, physicians’ offline reputation, as indicated by the title and the affiliated hospital ranking, does not rank high in the ML algorithms and does not appear in the top three levels of the tree structure. These physician offline reputation features are frequently employed by previous studies in similar contexts [7,15]. Although our LR results exhibit significant coefficients for these offline reputation features, in the tree structure, they only play a role in combination with other features in the lower levels. It is likely that patients experience different stages of awareness and learning during the phases of physician selection, free service, and paid service [9,34]. Although physician reputation may increase patients’ initial service awareness and influence physician selection, it seems that service experience (ie, service quality and intensive involvement) is a more important payment differentiator. Thus, our results show that regression may not be the best method to detect the impacts of various predictors and may yield oversimplified interpretation—regression only shows a linear additive relationship and excludes collinearity, whereas in reality, complex interactions and multiple paths to payment may exist.

In contrast to previous results that show the positive influence of prior physician-patient social ties on payment [18], our results show that a prior offline relationship with the physician does not always seem to be a facilitating factor for online payment. Although one subgroup of offline patients with existing social ties with the physician exhibits interactive service experiences and makes online payments, another offline subgroup seems to only use free services for simple follow-ups without deepening the online portion of the relationship and thus avoiding payment. Thus, it may be difficult for patients to completely shift their health care practices and habits from the offline to the online setting.

Previous studies also highlight virtual gifts as a positive signal for payment [2,12]. However, our findings suggest that virtual gifts may be a double-edged sword. For patients who have no prior offline connections with the physician, allowing them to show gratitude with a virtual gift function may not be a good strategy, as this type of patient may substitute this virtual gift for payment. However, if the service is intensive, virtual gifts and payment will be additive rather than substitutive.

In line with previous literature on online service delivery, responsive service is a key antecedent of payment [35,36]. Encouraging patient engagement (eg, encouraging multiple timely interactions with the physician) may help promote payment. As each response to the physician counts as one free trial for the patients, reluctance to consult further may arise at the end of each conversation turn. Persuading patients to keep on responding in a timely manner should be beneficial for establishing long-term patient-physician collaboration and attracting payments.

Previous studies in similar contexts generally use a linear regression approach; however, we employ ML—with its ability to mine massive fine-grained behavior data [37]—to explore the associations and predictive power of various consultation service–related features. The various classifiers based on different ML philosophies for a binary classification problem provide complementary views of how the model can help us understand payment. The feature ranking and configuration results from four ML approaches indicate that these features are not generating linear impacts, a finding that was not evident in previous studies.

### Conclusions

Online delivery of health care services is increasingly common and gives patients a new channel and expanded options for accessing health care services. However, many online medical consultation platforms are struggling to attract and retain patients who are willing to pay, and health care providers on the platform have the additional challenge of standing out in a crowd of physicians who can provide comparable services. This study explores the key features that contribute to patient payment in the online health care consultation market. By mining massive consultation data using ML approaches, our results show that features related to service delivery quality (eg, consultation dialog intensity and physician response rate), patient source (eg, online vs offline returning patients), and patient involvement (eg, provide social returns and reveal previous treatment) appear to contribute more to the patient’s payment decision than features related to physician reputation. We further identified five key feature configurations to help classify different interaction patterns between patients and physicians, which result in different payment outcomes.

## Appendix

Multimedia Appendix 1 Background information.

Multimedia Appendix 2 Data cleaning and analysis pipeline.

Multimedia Appendix 3 Data-driven feature selection.

Multimedia Appendix 4 Additional analysis results.

## Abbreviations

AUC: area under the receiver operating characteristic curve
DT: decision tree
GB: gradient boost
LR: logistic regression
ML: machine learning
RF: random forest
