# Supplementary material for: Analysis of Massive Online Medical Consultation Service Data to Understand Physicians’ Economic Return: Observational Data Mining Study
Source: JMIR Med Inform. 2020 Feb 18;8(2):e16765. doi: 10.2196/16765 (PMC7055801; doi:10.2196/16765)
Supplement: Multimedia Appendix 1 [file medinform_v8i2e16765_app1.docx]

## Multimedia Appendix 1: Background Information

**Table of Content**

| Illustration - Free and Payment Business Rules | Appendix 1 p. 2 |
| --- | --- |
| Illustration - Consultation History and Service Tag Classification | Appendix 1 p. 2 |
| Figure 1.1- A Screenshot of Consultation History between Patient i and Physician j | Appendix 1 p. 3 |
| Figure 1.2 - Screenshot of Transaction Records and Service Tags | Appendix 1 p. 3 |
| Table 1.1 - Types of patients and services based on the tags and conversation turns between patients and physicians. | Appendix 1 p. 4 |
| Table 2.1 - Summary Statistics of Key Features for Preprocessed Data | Appendix 2 p. 1 |
| Table 2.2 Description of Data Cleaning and Methodological Steps | Appendix 2 p. 2 |
| Table 2.3 Machine Learning Classifier Comparison | Appendix 2 p. 4 |
| Table 2.4 Evaluation Measures and Explanation | Appendix 2 p. 5 |
| Table 2.5. Hyperparameter Selection and Tuning Results | Appendix 2 p. 6 |
| Illustration - Feature Selection Procedure | Appendix 3 p. 1 |
| Table 3.1 Feature Selection Results | Appendix 3 p. 2 |
| Table 3.2 Correlation Matrix | Appendix 3 p. 4 |
| Illustration - Additional Analysis with Balanced Data | Appendix 4 p. 1 |
| Table 4.1 Model Performance for Balanced Data | Appendix 4 p. 1 |
| Table 4.2. Feature Importance Based on Balanced Data | Appendix 4 p. 2 |
| Figure 4.1 A Decision Tree Based on Balanced Data | Appendix 4 p. 3 |
| Illustration - Comparing Areas with Rich versus Few Healthcare Resources | Appendix 4 p. 4 |
| Table 4.3. Model Performance Comparison between Areas with Balanced Data | Appendix 4 p. 5 |
| Table 4.4. Feature Importance Comparison between Areas with Balanced Data | Appendix 4 p. 6 |
| Figure 4.2 A Decision Tree with Balanced Data for Remote Areas with Few Healthcare Resources | Appendix 4 p. 7 |
| Illustration - Comparing the Ten-Year Model with a Four-Year Model | Appendix 4 p. 8 |
| Table 4.5 Model Performance for Balanced Four-Year Data | Appendix 4 p. 8 |
| Table 4.6 Feature Importance Based on Balanced Four-Year Data | Appendix 4 p. 9 |
| Illustration - Additional Analysis with Outliers | Appendix 4 p. 9 |
| Table 4.7 Model Performance for Balanced Data with Outliers | Appendix 4 p. 10 |
| Table 4.8 Feature Importance for Balanced Data with Outliers | Appendix 4 p. 10 |

**i) Free and Payment Business Rules**

The platform we used in the study follows a service model under which a patient can gain up to three sessions of free consultations (i.e., three free trials) with one particular physician through two channels: either have previous offline hospital visit experience with the same physician (i.e., returning patients) or allow the platform to assign a physician when the patient initiates the consultation (i.e., assigned patients). Patients have to pay for the premium under two conditions: (1) he/she used up the three free trials with a particular physician and wants additional service from that physician; (2) he/she selects a physician when initiating the consultation without any previous offline visits. In this latter case, the patients may or may not have experienced free services from other physicians. Switch costs exist when a patient changes a physician in order to get more free consultations, which includes extra time and effort to report healthcare history, describe symptoms, establish a relationship with the new physician, and learn a new collaboration style. The free service is based on asynchronous picture-and-text communication, which allows a physician to significantly delay giving a response when busy. Premium services are diverse and are supported by more synchronous communication such as instant messages and phone calls with various time combinations (e.g., unlimited messages within 48 hours, monthly subscription, 15-minute phone call). Since the platform does not consistently provide visible tags for some premium services, in our data we only include premium services for which visible payment tags are consistently provided (i.e., 48-hour unlimited text & picture consultation, one-time phone-based consultation, one-question-and-one-answer) and exclude premium services where the data is less clear (i.e., services involve expert-team consultation and long-term subscriptions). In addition, our data includes free services as indicated by a “check-in” tag.

**ii) Consultation History and Service Tag Classification**

In our data, each record is a consultation history that includes picture-and-text based dialogues and service purchase records between patient *i* and physician *j* (see an example screenshot in Figure 1.1). As mentioned, certain patients have free trials on the platform. One free trial includes one conversation between the patient and the physician. One complete conversation is one turn by the patient (which can include multiple posts) followed by one turn by the physician (which can also include multiple posts). At the time of our data collection, the website provides visible tags to indicate whether the record includes payment (see Figure 1.2). In addition to the basic rules mentioned above, a physician may give a patient additional free consultation (in most cases, one or two free trials), but the platform does not encourage this practice and the physician needs to apply so that the platform gives the additional free services. Also, some paid services do not provide a tag (e.g., family doctor package). Each record can have more than one tag.

We coded these records to construct the paid versus free classes for the purpose of ML model training and validation through these service tags as well as the turns of conversations as per the free-trial rules. Table 1.1 presents different types of patient-physician relationships, as well as the identification criteria for free and paid classes.

Figure 1.1 A Screenshot of Consultation History between Patient *i* and Physician *j*

| Consultation history between patient *i* and physician *j*  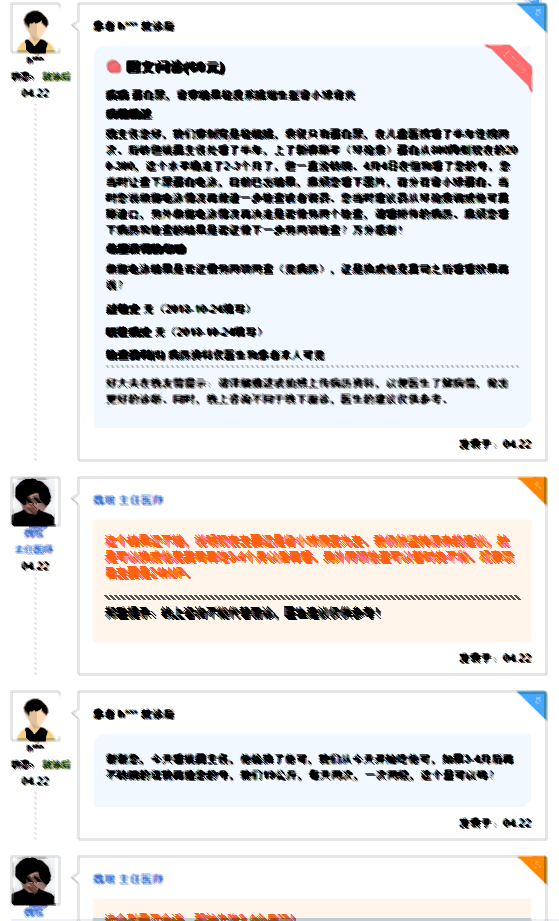  ***Converastion 1***  ***Conversation 2***  *Patient i*  *Status: have prior diagnosis*  *Post date: April 22*  *Picture-and-text service (60 Yuan)*   - *Disease/symptom description* - *Key question* - *Allergy* - *Past medical history* - *Previous diagnosis/ treatment documents*   *Physician j*  *Title: Chief physician*  *Answer date: April 22*  *Initial diagnosis and recommendation*  *Patient i - Further questions*  *Physician j – Additional diagnosis and recommendations* |
| --- |

Figure 1.2. Screenshot of Transaction Records and Service Tags

| 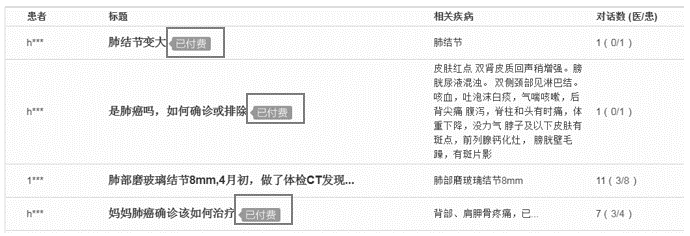  *Payment tags*  *Number of dialogues within a consultation history record*  *[total (patient/physician)]*  *Patient list*  *Consultation title*  *Disease type* |
| --- |

Table 1.1 Types of patients and services based on the tags and conversation turns between patients and physicians

|  | Free-trial-only | With premium subscription |
| --- | --- | --- |
| Have an offline relationship | (1) Returning patients  All offline return patients can enjoy three free trials with the same physician who gave them offline services.  These patients usually use this opportunity to clarify the unsolved issues during their offline hospital visit or for follow-ups  **Identification**: With a “check-in” tag/ No “payment” tag | (2) Returning patients who pay for the online premium service  If a returning patient wants to continue the service with a particular physician after using up the free trials, he/she has to pay for the premium.  **Identification:** With a “check-in” tag/ With at least one “payment” tag |
| No offline relationship | (3) Platform-assigned patients  If a patient does not select a physician (i.e., the physician is assigned by the platform) when he/she initiates the consultation, the patient enjoys three initial free trials.  These patients may have tried the free service from other physicians and switched to the current one or may be new to the platform.  **Identification:** No “check-in” tag/  No “payment” tag/ Turns of conversation less than four* | (4) Online patients who pay for the premium service  Two types of patients exist:  1. The patient who did not select the physician and used up the free trials must pay if he/she wants to obtain further services from that physician.  2. A patient who selects the physician has to pay (no free trials) when he/she initiates the consultation.  **Identification**: No “check-in” tag/  With at least one “payment” tag |

** Note.* Some physicians may decide to give extra free consultations (beyond three) to certain patients. These extra free consultations are not indicated by a tag on the system. To ensure that these special cases (which cannot be consistently identified) are removed from our data, all type 3 services (Platform-assigned patients) who had four or more free consultations were removed from the dataset.
