# Supplementary material for: Analysis of Massive Online Medical Consultation Service Data to Understand Physicians’ Economic Return: Observational Data Mining Study
Source: JMIR Med Inform. 2020 Feb 18;8(2):e16765. doi: 10.2196/16765 (PMC7055801; doi:10.2196/16765)
Supplement: Multimedia Appendix 2 [file medinform_v8i2e16765_app2.docx]

## Multimedia Appendix 2: Data Cleaning and Analysis Pipeline

| Table 2.1 Summary Statistics of Key Features for Preprocessed Data | | | | | | | |
| --- | --- | --- | --- | --- | --- | --- | --- |
|  | Mean (SD) | | | | Min – Max | | |
|  | All | Free-only | Paid | p-value* | All | Free-only | Paid |
| **Physician-related** | | | | | | | |
| Hospital ranking 1 | 0.947 (0.224) | 0.943 (0.232) | 0.955 (0.207) | 1.336 | 0 – 1 | 0 – 1 | 0 – 1 |
| Hospital ranking 2 | 0.022 (0.145) | 0.025 (0.156) | 0.014 (0.119) | <0.001 | 0 – 1 | 0 – 1 | 0 – 1 |
| Physician title 1 | 0.465 (0.499) | 0.434 (0.496) | 0.533  (0.499) | <0.001 | 0 – 1 | 0 – 1 | 0 – 1 |
| Physician title 2 | 0.326  (0.469) | 0.342 (0.474) | 0.291 (0.454) | <0.001 | 0 – 1 | 0 – 1 | 0 – 1 |
| Hospital location | 0.937  (0.243) | 0.925 (0.263) | 0.962 (0.191) | <0.001 | 0 – 1 | 0 – 1 | 0 – 1 |
| Physician tenure (month) | 74.724 (35.836) | 71.984 (36.111) | 80.782 (34.456) | <0.001 | 0 – 125 | 0 – 125 | 0 – 125 |
| Service intensity | 54.089 (48.951) | 52.423 (47.949) | 57.772 (50.904) | <0.001 | 0.008 – 207.062 | 0.008 – 207.062 | 0.009 – 207.062 |
| **Patient-related** | | | | | | | |
| PriorExam | 0.187  (0.39) | 0.06 (0.238) | 0.468 (0.499) | <0.001 | 0 – 1 | 0 – 1 | 0 – 1 |
| Private | 0.077 (0.267) | 0.066 (0.249) | 0.101 (0.301) | <0.001 | 0 – 1 | 0 – 1 | 0 – 1 |
| Offline connection | 0.714 (0.452) | 0.873 (0.332) | 0.363 (0.481) | <0.001 | 0 – 1 | 0 – 1 | 0 – 1 |
| **Service-related** | | | | | | | |
| Service duration (day) | 66.749 (204.027) | 55.374 (190.328) | 91.898 (229.458) | <0.001 | 1 – 4,297 | 1 – 4,297 | 1 – 3,824 |
| Total dialogue | 7.444 (6.379) | 6.268 (5.026) | 10.044 (8.061) | <0.001 | 1 – 35 | 1 – 31 | 1 – 35 |
| Patient posts | 5.792 (5.104) | 5.052 (4.384) | 7.429 (6.104) | <0.001 | 1 – 28 | 1 – 28 | 1 – 28 |
| Physician posts | 1.651 (1.776) | 1.215 (1.111) | 2.616 (2.46) | <0.001 | 0 – 7 | 0 – 3 | 0 – 7 |
| Response rate | 0.187 (0.163) | 0.179 (0.158) | 0.203 (0.173) | <0.001 | 0 – 0.875 | 0 – 0.75 | 0 – 0.875 |
| Question frequency | 1.195 (1.428) | 1.196 (1.238) | 0.923 (1.071) | <0.001 | 0 – 5.5 | 0 – 5.5 | 0 – 5.5 |
| Answer frequency | 0.222 (0.331) | 0.24 (0.345) | 0.185 (0.294) | <0.001 | 0 – 1.25 | 0 – 1 | 0 – 1.25 |
| Social return | 0.179 (0.383) | 0.179 (0.383) | 0.178 (0.383) | 0.263 | 0 – 1 | 0 – 1 | 0 – 1 |

*Mean difference between free and paid. Levene’s test indicates that the variances are not equal between free-trial-only and paid groups across all variables.

| Table 2.2 Description of Data Cleaning and Methodological Steps | |
| --- | --- |
| Methodological Steps | Purposes |
| 1. Data extraction, cleaning, and screening | Extract appropriate variables from the scraped dataset based on the key predictive features discussed above. |
|  | Transform the raw data into appropriate data representations to ensure the quality of inputs and outcomes. This process includes:   - Extract numeric value from string format data - Transform categorical data into dummies - Remove records with missing values - Detect and remove outliers (based on 95% quantile as the threshold) - Prepare the outcome variable: turn string format tags into a categorical variable |
| 2. Data-driven feature selection | Dimension reduction: retain the most useful information to generate a parsimonious prediction model that can achieve good classification performance. Decision Tree algorithm is used. Dimension reduction techniques include:   - Low variance filtering - High correlation filtering - Backward feature elimination - Forward feature construction |
| 3. Model training and validation  3.1 Classification algorithm selection  3.2 Hyperparameter tuning & model training  3.3 Model validation | Execute the training and validation tasks in loops to optimize the hyperparameters, find the model parameters and compare the performance of different ML algorithms. The following steps are executed iteratively in loops.  A variety of common algorithms are used to ensure the accessibility of our approach. We applied Decision Tree, Logistic Regression, Random Forest, and Gradient Boosting in the main analysis, and AdaBoost and XGBoost in the additional analysis to represent different machine learning philosophies for classification problems.  Determine the optimal setting of the ML algorithm in order to achieve better performance during model training and prediction. To account for the overfitting issue, we used a 10-fold cross-validation approach.  Test the model (i.e., the feature configurations) by predicting the payment outcomes in the validation set. |
| 4. Model evaluation | The performance of the predictive models with the best hyperparameters is evaluated based on commonly accepted metrics: recall, specificity, precision, F-measure, accuracy, balanced accuracy, and AUC of ROC. |
| 5. Feature importance | Identify the relative importance of the service features based on the indicators given by the ML classifiers: (1) regression coefficients provided by logistic regression classifier, (2) the level of splits provided by decision tree, (3) feature importance score provided by random forest and gradient boost algorithms (detailed explanation of the calculation can be found on http://docs.h2o.ai/h2o/latest-stable/h2o-docs/variable-importance.html#).  The decision tree algorithm gives extra information on feature hierarchies which indicate the configurations of the important features that contribute to free consultation or payment. |

| Table 2.3 Machine Learning Classifier Comparison | | | |
| --- | --- | --- | --- |
| ML Algorithm | Classifier System | Parametric | Explanation |
| *Decision tree (DT) | Single | No | Find the decision boundary by bisecting the space into hyper-rectangles. |
| *Logistic regression (LR) | Single | Yes | Find a single line decision boundary (linear or not linear) using Sigmoid function. |
| *Random forest (RF) | Multiple (bagging) | No | Decision tree + Bagging:  Trees are grown in parallel by randomly selecting samples (bootstrapping) with a subset of features that can give the best split on the training data. Predictions are aggregated from these sub-models. |
| *Gradient Boost tree (GB) | Multiple (boosting) | No | Decision tree + Boosting:  Instead of updating the data distribution to highlight the misclassified observations, the gradient boosting approach focuses on the residuals and uses a gradient descent method to minimize the loss function. |
| AdaBoost tree (ADA) | Multiple (boosting) | No | Decision tree + Boosting:  Bootstrap data and grow trees sequentially by iteratively updating data distribution with up- weighted previously misclassified data (i.e., previously misclassified observations are more likely to appear in the next bootstrap) |
| XGBoost tree (XG) | Multiple (boosting) | No | Gradient boosting technique being optimized for speed and performance (i.e., apply penalties when updating trees and residuals). |

***Note:*** The classifiers with an asterisk are used in main analysis, whereas the others are used in additional analyses for model performance verification. These classifiers differ in their classification approaches. For example, Logistic Regression is a parametric approach that makes prior assumptions on data distribution and the form of mapping functions (e.g., a linear function with a fixed number of parameters), whereas the others are non-parametric, so the complexity of the model may grow as more training data is input into the system. In addition, the random forest and boosted trees are ensemble learning approaches (i.e., using multiple classifiers in the system). However, they differ in learning approaches (i.e., bagging vs. boosting), and AdaBoosting and Gradient Boosting (also XGBoost) differ in boosting approaches (i.e., focus on data distribution vs. residuals). These differences reflect different ML logic and decision boundary detection approaches.

| Table 2.4 Evaluation Measures and Explanation | | |
| --- | --- | --- |
| Measure | Calculation | Explanation |
| Precision | TP / (TP + FP) | The positive predictive value (also called purity). It shows the percentage of samples correctly labeled as positive (i.e., *paid*) out of all predicted positive. |
| Recall | TP / (TP + FN) | The true positive rate (also called sensitivity). It shows the percentage of samples correctly labeled as positive out of all true positives. |
| Specificity | TN / (TN + FP) | The true negative rate (also called selectivity). It shows the percentage of samples correctly labeled as negative out of all true negatives (i.e., *free*). |
| F-measure | 2* Precision * Recall  Precision + Recall | The harmonic mean of precision and recall. It is useful for unbalanced data since it takes into account the cost of both false negatives and false positives. |
| Balanced accuracy | (Recall + Specificity)/2 | The average of recall and specificity. It combines correctly predicted positives and correctly predicted negatives. |
| Area under the ROC curve (AUC) | P (X_+_ > X_-_)  X+: the score of a random positive sample  X-: the score of a random negative sample | AUC represents the probability that the ML model ranks a random true positive sample more highly than a random true negative sample (i.e., true positive rate vs. false positive rate). The closer to 1, the better the prediction. AUC=1 means 100% true positive and 0% false positive. |

***Note.*** TP – true positive; FP – false positive; FN – false negative; TN – true negative.

| Table 2.5. Hyperparameter Selection and Tuning Results | | | |
| --- | --- | --- | --- |
| **Hyperparameters** | **Optimal Value** | **Explanation** | **Other Default Configurations** |
| **(Simple) decision tree*** | | | |
| Minimum leaf size  [2-10] | 2 | It defines the minimum sample needed in a leaf node, which is used to control overfitting. | - The algorithm follows the C4.5 program  - pruning: enabled (MDL method)  - reduced error pruning: enabled  - quality measure: Gini index  - average split point (the split value for numeric features): the mean value of two adjacent feature values  -tree depth: unlimited |
| Minimum node size  (min_leaf_size *2) | 4 | The minimum number of samples required to split a node. This is a stopping criterion; if the sample size is smaller this number, the tree will not grow further. |  |
| **Logistic regression*** | | | |
| Prior distribution (uniform, Gauss or Laplace) | Gauss | The assumption of coefficient distribution which is related to regularization (i.e., the technique to shrink the learned regression coefficient towards zero to avoid overfitting).  Uniform: no regularization  Gauss: the coefficients are assumed to be normally distributed, which is equivalent to using L2 regularization.  Laplace: the coefficients are assumed to follow a Laplace distribution, which is related to L1 regularization. | - algorithm: stochastic average gradient (follows Schmidt et al. 2016)  - learning rate: 0.1  - epsilon (determine whether the model converge): 1e-5 |
| Variance of prior distribution (*var*)  [0.01, 0.05, 0.1] | 0.1 | Controls the degree of regularization. The regularization coefficient $\lambda=\frac{1}{var}$. Thus, the larger the variance, the less the regularization. |  |
| Epochs | 10000 | The maximum number of learning iterations. (The algorithm stops when it reaches convergence) |  |
| **Random forest*** | | | |
| Number of trees in the forest [50,300] | 50 | Including more trees is better to learn the data, but it significantly slows down the training process. | - minimum relative improvement rate: 0.00001  - sample rate per tree: 1  - class-specific sample rate per tree: 1  - feature sampling rate per tree: 1  - feature sampling rate per split: 1  - the algorithm follows Breiman et al.’s (1984) description. H2O framework is implemented in our analysis. |
| Max number of levels in each decision tree  (tree depth) [2,6] | 6 | The more splits a tree has, the more information can be captured from the data, but a higher depth means the algorithm learns the relations at a very specific level (i.e., overfitting). |  |
| Minimum number of observations in a leaf (min leaf size) [2-10] | 2 | It defines the minimum sample needed in a leaf, which is used to control overfitting. |  |
| **AdaBoost tree*** | | | |
| Min leaf size [2-10] | 8 | The minimum sample needed in a leaf node. | - number of models to learn: 10  - Other default decision tree settings  - The algorithm follows Freund & Schapire (1997) |
| **Gradient Boost tree** | | | |
| Number of trees  [≤100, ≤200, ≤300] | 100 | Specifies the optimal number of trees to grow in the model. | - maximum tree depth: 10  - minimum observations for a leaf: 10  - loss function: auto  - random seed: yes  - - minimum relative improvement rate: 0.00001  - sample rate per tree: 1  - class-specific sample rate per tree: 1  - feature sampling rate per tree: 1  - feature sampling rate per split: 1  - the implemented algorithm follows Friedman (2001) |
| Learning rate  [0.05, 0.1, 0.3, 0.5] | 0.1 | The scalar that determines the size of each learning step. A lower learning rate requires more trees to achieve the same level of fit. |  |
| **XGBoost tree** | | | |
| Eta  [0.01, 0.05, 0.1, 0.3, 0.5] | 0.3 | Similar to the concept of learning rate in gradient boosting. | - the minimum sum of observation weight in a node: 1  - maximum delta step: 0 (no constraint)  - subsampling rate: 1  - feature sampling rate per tree: 1  - feature sampling rate per split: 1  - L2 regularization (lambda): 1  - L1 regularization (alpha): 1  - tree construction method: auto  - grow policy: split nodes closest to the root  - the algorithm implemented follows the official document of XGBoost. |
| Gamma [0-10] | 1 | The minimum loss reduction required to make a further split of a tree |  |
| Tree depth [2-10] | 6 | The maximum depth of a tree. |  |
| Boosting rounds  [50, 100, 200] | 50 | Rounds of boosting iteration. |  |

****Note:*** The classifiers with an asterisk are used in main analysis, whereas the others are used in additional analyses for model performance verification.

References:

Breiman, L., Friedman, J., Stone, C. J., & Olshen, R. A. (1984). Classification and Regression Trees. CRC Press.

Freund, Y., & Schapire, R. E. (1997). A decision-theoretic generalization of on-line learning and an application to boosting. Journal of computer and system sciences, 55(1), 119-139.

Friedman, J. H. (2001). Greedy function approximation: a gradient boosting machine. Annals of Statistics, 1189-1232.

Schmidt, M., Le Roux, N., & Bach, F. (2017). Minimizing finite sums with the stochastic average gradient. Mathematical Programming, 162(1-2), 83-112.

XGBoost official documentation. Access from https://xgboost.readthedocs.io/en/latest/parameter.html
