# Supplementary material for: Analysis of Massive Online Medical Consultation Service Data to Understand Physicians’ Economic Return: Observational Data Mining Study
Source: JMIR Med Inform. 2020 Feb 18;8(2):e16765. doi: 10.2196/16765 (PMC7055801; doi:10.2196/16765)
Supplement: Multimedia Appendix 3 [file medinform_v8i2e16765_app3.docx]

## Multimedia Appendix 3: Data-Driven Feature Selection

Retaining useful features is an essential step in ML and improves computation efficiency without over sacrificing classification task performance. Although we expect all the 18 initial features to be useful, some of them may have low explanatory value and high multicollinearity, rendering them less useful. To make the model more parsimonious, we follow Silipo et al.’s (2015) approach and run four tests – low variance filtering, high correlation filtering, backward feature selection, and forward feature selection – to eliminate less useful features before formal model building. The first two are filter approaches in which the features are evaluated based on data characteristics (i.e., variance and correlation), and the last two are wrapper approaches, which use ML algorithms and statistical re-sampling techniques to decide the importance of the features (Yu and Liu 2003). We use a decision tree classifier, which is a classic ML algorithm for feature selection (Dash and Liu 1997). Since the objective at this stage is not to assess or improve classification performance but to find a baseline for model training and selection, we do not perform hyperparameter tuning at this stage.

Before performing the feature selection procedure, we train and validate the data with decision tree algorithm using a 10-fold cross-validation approach with all the features (i.e., using 18 features as input). The purpose is to set the baseline level for classification performance so that we can compare the performance of various feature selection approaches and balance the feature retention options. The decision tree algorithm reaches a balanced accuracy of 95.9% (F-measure=0.965, Recall= 0.959, Precision= 0.971).

Then we run the models iteratively with four feature selection techniques. First, dimensions with very low variance (i.e., almost a constant value) can be eliminated since they contain little information to discriminate the classes. To find the optimal threshold for elimination, we apply the decision tree algorithm in loops with 10-fold cross-validation, and the threshold that can maximize the balanced accuracy score is selected for deciding the feature retention. As shown in Table 3.1, a variance of 2% is considered as a low-bound threshold, and if dimension reduction is executed based on this threshold, after eliminating the low variance features, the classification (balanced) accuracy can reach 97.85%.

Second, highly correlated dimensions can be eliminated since they contain similar information. Thus, removing one of the two highly correlated dimensions can simplify the input space without sacrificing the predictive power for the future. Pairs of correlations are calculated after variable normalization since the calculation of the correlation coefficient largely depends on the data range, and one of the two highly correlated dimensions is eliminated if it goes above the correlation threshold. Similar to the identification of low variance threshold, the optimal correlation threshold is decided by running a loop of training and validation tests with three algorithms to achieve the highest classification accuracy. The correlation matrix is presented in Table 3.2. The optimal threshold for dimension reduction decided by the algorithm is 0.3. Five features are retained (see Table 3.1) based on this threshold, which can yield a balanced accuracy level of 78.1% in the classification task on the original data based on the Decision Tree algorithm. However, the algorithm-decided feature retention may be too strict, resulting in too much information lost (i.e., only the most parsimonious solution is given). Thus, we try a manual procedure in order to retain more feature information. We manually select features with low correlations (below 0.3) based on the correlation table and input them all together into the training model. Ten features were used which can yield a balanced accuracy of 97.86%.

Third, backward feature elimination removes one input feature at one time during the training and validation loop, and each feature removal should minimize the increase of error rate in the classification performance. When setting the expected accuracy threshold for feature retaining at 96% (the baseline balanced accuracy for decision tree algorithms), five features are retained as the most parsimonious solution that yields a 97.8% balanced accuracy.

Fourth, contrary to the backward approach, forward feature selection starts from inputting one feature and adds one new feature at a time in the iterations to obtain the best classification accuracy. Similar to the backward approach, when we set the target accuracy threshold at 96%, four features are selected as the most parsimonious solution. The prediction on the validation set yields a 97.8% balanced accuracy level as well.

The four approaches listed above did not give us consistent feature selection results, which is within our expectation because they follow different philosophies to eliminate the features. Since forward and backward approaches gave us the most parsimonious solutions, we decide to retain additional features that are selected by both low variance filtering and one of the high correlation filtering approaches. Consequently, in response to our first research objective regarding which key features of online medical consultation services are associated with patient payment, our feature selection analysis suggests that the key features are: *ranking 2, title 1, PriorExam, private, offline connection, total dialogue, patient posts, response rate, question frequency, answer frequency, and social retur*n. These features are used as the inputs in the analyses which follow.

| Table 3.1 Feature Selection Results | | | | | |
| --- | --- | --- | --- | --- | --- |
|  | Low Variance filtering | High correlation filtering | High correlation filtering (manual) | Backward feature elimination | Forward feature elimination |
| Hospital ranking 1 | √ |  |  |  |  |
| Hospital ranking 2 | √ | √ | √ |  |  |
| Physician title 1 | √ | √ | √ |  |  |
| Physician title 2 | √ |  |  |  |  |
| Hospital location | √ |  |  |  |  |
| Physician tenure (month) | √ |  |  |  |  |
| Service intensity | √ |  |  |  |  |
| PriorExam | √ |  | √ |  |  |
| Private | √ | √ | √ | √ | √ |
| Offline connection | √ |  | √ | √ | √ |
| Service duration (day) |  |  | √ |  |  |
| Total dialogue | √ | √ |  |  |  |
| Patient posts | √ |  | √ | √ | √ |
| Physician posts | √ |  |  |  |  |
| Response rate | √ |  | √ | √ |  |
| Question frequency | √ |  | √ |  |  |
| Answer frequency | √ | √ |  |  |  |
| Social return | √ |  | √ | √ | √ |
| *Balanced accuracy* | *97.85%* | *78.13%* | *97.86%* | *97.8%* | *97.8%* |
| *Threshold* | *0.02* | *0.3* | *0.3* | *-* | *-* |

| Table 3.2 Correlation Matrix | | | | | | | | | | | | | | | | | | |
| --- | --- | --- | --- | --- | --- | --- | --- | --- | --- | --- | --- | --- | --- | --- | --- | --- | --- | --- |
|  | V1 | V2 | V3 | V4 | V5 | V6 | V7 | V8 | V9 | V10 | V11 | V12 | V13 | V14 | V15 | V16 | V17 | V18 |
| 1-Title1 | 1.000 |  |  |  |  |  |  |  |  |  |  |  |  |  |  |  |  |  |
| 2-Title2 | -0.648 | 1.000 |  |  |  |  |  |  |  |  |  |  |  |  |  |  |  |  |
| 3-Ranking1 | 0.053 | 0.010 | 1.000 |  |  |  |  |  |  |  |  |  |  |  |  |  |  |  |
| 4-Ranking2 | -0.076 | 0.013 | -0.625 | 1.000 |  |  |  |  |  |  |  |  |  |  |  |  |  |  |
| 5-Physician Tenure | 0.350 | -0.072 | 0.108 | -0.146 | 1.000 |  |  |  |  |  |  |  |  |  |  |  |  |  |
| 6-Total Dialogue | -0.038 | 0.051 | 0.026 | -0.015 | -0.032 | 1.000 |  |  |  |  |  |  |  |  |  |  |  |  |
| 7-PatientPosts | -0.025 | 0.054 | 0.032 | -0.020 | -0.020 | 0.977 | 1.000 |  |  |  |  |  |  |  |  |  |  |  |
| 8-PhysicianPosts | -0.064 | 0.027 | 0.001 | 0.004 | -0.057 | 0.786 | 0.634 | 1.000 |  |  |  |  |  |  |  |  |  |  |
| 9-SocialReturn | 0.032 | 0.002 | 0.006 | -0.020 | 0.081 | 0.254 | 0.233 | 0.242 | 1.000 |  |  |  |  |  |  |  |  |  |
| 10-Service Duration | 0.037 | -0.017 | 0.010 | -0.007 | 0.085 | 0.238 | 0.234 | 0.181 | 0.098 | 1.000 |  |  |  |  |  |  |  |  |
| 11-Response Rate | -0.063 | -0.003 | -0.040 | 0.035 | -0.038 | 0.253 | 0.092 | 0.642 | 0.099 | 0.063 | 1.000 |  |  |  |  |  |  |  |
| 12-Question Frequency | -0.007 | 0.006 | 0.016 | -0.014 | -0.029 | -0.206 | -0.148 | -0.316 | -0.180 | -0.280 | -0.311 | 1.000 |  |  |  |  |  |  |
| 13-Service Intensity | 0.113 | -0.049 | 0.012 | -0.010 | 0.095 | 0.116 | 0.120 | 0.071 | -0.016 | 0.045 | 0.052 | 0.064 | 1.000 |  |  |  |  |  |
| 14-Offline Connection | -0.205 | 0.103 | -0.004 | 0.035 | -0.300 | 0.151 | 0.153 | 0.102 | -0.133 | -0.076 | 0.006 | 0.035 | -0.080 | 1.000 |  |  |  |  |
| 15-Status1 | 0.155 | -0.077 | 0.003 | -0.033 | 0.197 | -0.115 | -0.115 | -0.081 | 0.075 | -0.011 | -0.028 | 0.039 | 0.036 | -0.681 | 1.000 |  |  |  |
| 16-Status2 | 0.047 | -0.032 | -0.005 | 0.006 | 0.103 | -0.022 | -0.036 | 0.026 | -0.073 | 0.152 | 0.134 | -0.123 | 0.104 | -0.298 | -0.139 | 1.000 |  |  |
| 17-Answer Frequency | -0.065 | 0.016 | -0.020 | 0.013 | -0.053 | 0.018 | -0.040 | 0.179 | -0.052 | -0.195 | 0.498 | 0.376 | 0.051 | 0.041 | 0.008 | -0.008 | 1.000 |  |
| 18-Location | 0.028 | -0.018 | 0.033 | -0.108 | 0.061 | 0.006 | 0.012 | -0.013 | 0.018 | 0.009 | -0.035 | 0.007 | 0.053 | -0.096 | 0.074 | 0.049 | -0.029 | 1.000 |

**References**

Dash, M., and Liu, H. 1997. "Feature Selection for Classification," *Intelligent data analysis* (1:1-4), pp. 131-156.

Silipo, R., Adae, I., Hart, A., and Berthold, M. 2015. "Seven Techniques for Data Dimensionality Reduction." Retrieved May 1, 2019, from https://www.knime.com/blog/seven-techniques-for-data-dimensionality-reduction

Yu, L., and Liu, H. 2003. "Feature Selection for High-Dimensional Data: A Fast Correlation-Based Filter Solution," *Proceedings of the 20th international conference on machine learning (ICML-03)*, pp. 856-863.
