# Supplementary material for: Analysis of Massive Online Medical Consultation Service Data to Understand Physicians’ Economic Return: Observational Data Mining Study
Source: JMIR Med Inform. 2020 Feb 18;8(2):e16765. doi: 10.2196/16765 (PMC7055801; doi:10.2196/16765)
Supplement: Multimedia Appendix 4 [file medinform_v8i2e16765_app4.docx]

## Multimedia Appendix 4: Additional Analysis Results

**Additional Analysis with Balanced Data**

We randomly selected 492,902 free-only transactions (because N_paid_=492,902) and repeated the whole procedures with three algorithms – logistic regression, decision tree, and random forest. The model performance and feature importance results are presented in Table 4.1 and 4.2. In general, the changes for all performance measures are minor. Logistic Regression and Random Forest improved recall at about 4.5%, indicating a reduction in type II error, whereas the improvement in the decision tree is negligible. The change in overall performance measures (i.e., balanced accuracy and AUC) is minor across all three algorithms. Feature importance ranking is very similar to the main analysis – offline connection, total dialogue, response rate, social return, prior exam and private are consistently ranked highly, whereas physician title, question frequency, and the second-tiered hospital ranking are consistently ranked low. However, the tree structure is much clearer for the balanced data (see Figure 4.1). In summary, features related to service quality and patient involvement seem to be more important than physician offline reputation (e.g., affiliation and title).

| Table 4.1 Model Performance for Balanced Data | | | | | | |
| --- | --- | --- | --- | --- | --- | --- |
|  | Logistic regression | | Decision tree | | Random forest | |
|  | Score | Change | Score | Change | Score | Change |
| Recall | 0.897 | +0.046 | 0.955 | +0.006 | 0.953 | +0.045 |
| Precision | 0.905 | +0.009 | 0.990 | +0.002 | 0.966 | -0.018 |
| Specifity | 0.906 | -0.050 | 0.991 | -0.004 | 0.968 | -0.025 |
| F-measure | 0.901 | +0.028 | 0.972 | -0.004 | 0.959 | +0.015 |
| Accuracy | 0.902 | -0.021 | 0.973 | -0.008 | 0.961 | -0.006 |
| Balanced accuracy | 0.902 | -0.001 | 0.973 | +0.001 | 0.961 | +0.010 |
| AUC | 1.000 | 0.000 | 0.988 | 0.000 | 0.989 | +0.001 |
| Optimal Hyperparameter | Prior distribution: Laplace  Variance of prior distribution = 0.1  Epochs=10,000 | | Minimum leaf size= 4 | | Number of trees in the forest= 250  Minimum leaf size = 6  Tree depth = 6 | |

| Table 4.2. Feature Importance Based on Balanced Data | | | |
| --- | --- | --- | --- |
|  | LR | DT | RF |
| 1 | Response rate  (-12.072) | Offline connection (1) | Offline connection (29.79%) |
| 2 | Offline connection  (-5.025) | Social return (2) | Total Dialogue  (19.14%) |
| 3 | Social return  (-2.563) | Total dialogue (2) | PriorExam  (16.20%) |
| 4 | Patient posts  (-2.457) | Private (3) | Response rate  (13.72%) |
| 5 | Total dialogue  (2.340) | Response rate (3) | Patient posts  (10.21%) |
| 6 | PriorExam  (1.701) | PriorExam (4) | Social return  (5.74%) |
| 7 | Private  (-0.682) | Question_frq (5) | Answer_frq |
| 8 | Answer_frq  (-0.283) | Answer_frq (5) | Private |
| 9 | Ranking 2  (-0.268) | Patient posts (6) | Question_frq |
| 10 | Question_frq  (-0.137) | Title 1 (7) | Title 1 |
| 11 | Title 1  (-0.099) | Ranking 2 (10) | Ranking 2 |

*Note.* LR-logistic regression. Coefficients are shown in the brackets. DT – (simple) decision tree. The numbers in the brackets show the highest level of tree splits; RF – random forest. The numbers in the brackets show the percentage feature importance (only those above 5% are shown).

| Figure 4.1 A Decision Tree Based on Balanced Data |
| --- |
|  |
|   Note: Only the top 3 levels are presented in the figure to illustrate the hierarchy and subgroups |
|  |

**Comparing Areas with Rich versus Few Healthcare Resources**

We randomly selected 81,311 *free* records from remote areas and 474,203 *free* records from resource-rich areas to balance the *paid* records. The results are presented in Table 4.3 and 4.4. The model performance scores are similar to those of the main analysis (as well as the analysis with balanced data) with slightly lower scores for the remote areas, indicating that the applicability of our model is not influenced by physicians’ location. However, although the overall feature importance ranking is similar to the main analysis (i.e., service quality features and patient involvement features rank much higher than physician reputation features), remote areas with few healthcare resources present quite different feature configurations according to the decision tree structure and logistic regression (see Figure 4.2). In general, the amount of total dialogue is the dominant feature that is associated with payment. For the consultations with fewer communication turns, patients with offline connections are less likely to pay, unless total dialogue and response rate are high. Social return may substitute payment as indicated by 73% of the patients who had no offline connections but provided social returns did not go beyond free services. However, the amount of patient posts only exhibits an important impact for those from remote areas with few healthcare resources. It may be due to the differences in medical consultation habits, or difficulties in building trust and commitment for those “less known” providers. More research needs to be done to explore the reason behind this difference.

| Table 4.3. Model Performance Comparison between Areas with Balanced Data | | | | | | | | | | | | |
| --- | --- | --- | --- | --- | --- | --- | --- | --- | --- | --- | --- | --- |
|  | Areas Rich in Healthcare Resources | | | | | | Areas with Few Healthcare Resources | | | | | |
|  | LR (balanced) | | DT (balanced) | | RF (balanced) | | LR (balanced) | | DT (balanced) | | RF (balanced) | |
|  | Score | Change | Score | Change | Score | Change | Score | Change | Score | Change | Score | Change |
| Recall | 0.898 | 0.002 | 0.956 | 0.001 | 0.954 | 0.001 | 0.928 | 0.031 | 0.948 | -0.007 | 0.959 | 0.006 |
| Precision | 0.906 | 0.000 | 0.991 | 0.000 | 0.967 | 0.002 | 0.926 | 0.021 | 0.995 | 0.004 | 0.957 | -0.009 |
| Specifity | 0.906 | 0.000 | 0.991 | 0.000 | 0.969 | 0.001 | 0.926 | 0.020 | 0.995 | 0.004 | 0.957 | -0.011 |
| F-measure | 0.902 | 0.001 | 0.973 | 0.000 | 0.961 | 0.001 | 0.927 | 0.026 | 0.971 | -0.002 | 0.958 | -0.001 |
| Accuracy | 0.902 | 0.001 | 0.973 | 0.000 | 0.961 | 0.001 | 0.927 | 0.026 | 0.971 | -0.002 | 0.958 | -0.003 |
| Balanced accuracy | 0.902 | 0.001 | 0.973 | 0.000 | 0.961 | 0.001 | 0.927 | 0.026 | 0.971 | -0.002 | 0.958 | -0.003 |
| AUC | 1.000 | 0.000 | 0.988 | 0.000 | 0.988 | 0.000 | 1.000 | 0.000 | 0.983 | -0.005 | 0.989 | 0.000 |

Note. N_rich_=1,482,554; N_few_=100,010; N_few-paid_ =81,311; N_rich-paid_=474,203

| Table 4.4. Feature Importance Comparison between Areas with Balanced Data | | | | | | |
| --- | --- | --- | --- | --- | --- | --- |
|  | Areas Rich in Healthcare Resources | | | Areas with Few Healthcare Resources | | |
|  | LR | DT | RF | LR | DT | RF |
| 1 | Response rate  (-12.02***) | Offline connection (1) | Offline connection (30%) | Response rate  (-11.09***) | Total dialogue (1) | Total Dialogue (32.5%) |
| 2 | Offline connection  (-5.11***) | Social return  (2) | Total Dialogue (18.6%) | Offline connection  (-4.12***) | Offline connection (2) | Offline  (17.4%) |
| 3 | Social return  (-2.69***) | Total dialogue (2) | Status1  (16.5%) | PriorExam (3.03***) | Response rate (2) | Response rate (16.8%) |
| 4 | Patient posts  (-2.45***) | Private  (3) | Response rate (13.6%) | Patient posts  (-2.74***) | Social return (3) | Patient posts (16.60%) |
| 5 | Total Dialogue (2.33***) | Response rate (3) | Patient posts (9.9%) | Total Dialogue (2.60***) | PriorExam  (4) | PriorExam  (9.7%) |
| 6 | Status1  (1.62***) | PriorExam  (4) | Social return  (6%) | Status2  (1.32***) | Patient posts (5) | Question_frq |
| 7 | Status2  (-0.81***) | Answer_frq  (6) | Answer_frq | Social return  (-0.92***) | Title 1  (6) | Answer_frq |
| 8 | Ranking2  (-0.35***) | Question_frq  (7) | Status2 | Title1  (-0.49***) | Private  (7) | Social return |
| 9 | Answer_frq  (-0.31***) | Title1  (7) | Question_frq | Ranking2  (-0.18) | Question_frq (8) | Private |
| 10 | Question_frq  (-0.13***) | Ranking2  (8) | Title1 | Answer_frq  (-0.06) | N/A | Title1 |
| 11 | Title1  (-0.07***) | Patient posts  (9) | Ranking2 | Question_frq  (-0.03) | N/A | Ranking2 |

*Note.* LR-logistic regression. Coefficients are shown in the brackets. DT – (simple) decision tree. The numbers in the brackets show the highest level of tree splits; RF – random forest. The numbers in the brackets show the percentage feature importance (only those above 5% are shown).

| Figure 4.2 A Decision Tree with Balanced Data for Remote Areas with Few Healthcare Resources |
| --- |
|   Note: Only top 3 levels are presented in the figure to illustrate the hierarchy and subgroups |

**Comparing the Ten-Year Model with a Four-Year Model**

In total, there are 1,090,041 consultation records after 2015 (N_free_=783,769 and N_paid_=306,272). We create a balanced dataset by randomly selecting 306,272 free-only records. The ML performance results and feature rankings are presented in Table 4.5 and 4.6. Logistic regression exhibits a better classification for the more recent data – there is an 8.5% improvement in recall, 7.1% improvement in F-measure, and 4.1% improvement in balanced accuracy – whereas decision tree has a minor reduction in performance. All three ML algorithms exhibit excellent overall classification performance. The feature ranking is consistent with the main analysis (as well as the other additional analyses) – total dialogue, offline connection, prior examination and response rate are ranked highly, and physician title and affiliation are ranked low. The amount of patient posts is a bit controversial since the decision tree algorithm ranked it low, whereas the other two algorithms ranked it highly. In general, the performance of our model is not influenced by potential systematic differences due to the market cycle.

| Table 4.5 Model Performance for Balanced Four-Year Data | | | | | | |
| --- | --- | --- | --- | --- | --- | --- |
|  | LR | | DT | | RF | |
|  | Score | Change | Score | Change | Score | Change |
| Recall | 0.936 | 0.085 | 0.954 | 0.004 | 0.954 | 0.046 |
| Precision | 0.951 | 0.055 | 0.989 | 0.000 | 0.974 | -0.010 |
| Specifity | 0.952 | -0.004 | 0.990 | -0.006 | 0.975 | -0.018 |
| F-measure | 0.944 | 0.071 | 0.971 | 0.002 | 0.964 | 0.019 |
| Accuracy | 0.944 | 0.021 | 0.972 | -0.009 | 0.964 | -0.002 |
| Balanced accuracy | 0.944 | 0.041 | 0.972 | -0.001 | 0.964 | 0.014 |
| AUC | 1.000 | 0.000 | 0.986 | -0.002 | 0.988 | 0.000 |

| Table 4.6 Feature Importance Based on Balanced Four-Year Data | | | |
| --- | --- | --- | --- |
|  | LR | DT | RF |
| 1 | Total Dialogue (61.25***) | Offline connection (1) | Offline connection (30%) |
| 2 | Patient post (-45.27***) | Social return (2) | Total Dialogue (20%) |
| 3 | Response rate (-10.21***) | Total dialogue (2) | Response rate (17.2%) |
| 4 | Offline connection (-5.71***) | Private (3) | PriorExam (17%) |
| 5 | PriorExam (2.09***) | Response rate (3) | Patient post (8.8%) |
| 6 | Social return (-1.43***) | PriorExam (4) | Private |
| 7 | Answer_frq (-0.71***) | Question_frq (5) | Social return |
| 8 | Private (0.36***) | Ranking 2 (7) | Answer_frq |
| 9 | Question_frq (0.15***) | Title 1 (8) | Question_frq |
| 10 | Ranking2 (-0.05) | Patient posts (9) | Title1 |
| 11 | Title 1 (0.006) | Answer_frq (9) | Ranking2 |

*Note.* LR-logistic regression. Coefficients are shown in the brackets. DT – (simple) decision tree. The numbers in the brackets show the highest level of tree splits; RF – random forest. The numbers in the brackets show the percentage feature importance (only those above 5% are shown).

**Additional Analysis with Outliers**

There are 1,691,491 consultation records available in total if outliers are included (N_paid_=545,134; N_free_=1,146,357). A balanced dataset is created using 545,134 randomly selected free-only records. The performance measures and feature importance rankings presented in Table 4.7 and 4.8 exhibit similar patterns with only minor changes from the main analysis. This indicates that the model with 11 features is robust to outliers (e.g., extreme cases and possible bad data points due to deficiencies in the web crawler).

| Table 4.7 Model Performance for Balanced Data with Outliers | | | | | | |
| --- | --- | --- | --- | --- | --- | --- |
|  | LR | | DT | | RF | |
|  | Score | Change | Score | Change | Score | Change |
| Recall | 0.905 | 0.054 | 0.965 | 0.016 | 0.931 | 0.023 |
| Precision | 0.906 | 0.010 | 0.993 | 0.004 | 0.969 | -0.015 |
| Specifity | 0.905 | 0.054 | 0.965 | 0.016 | 0.931 | 0.023 |
| F-measure | 0.905 | 0.032 | 0.979 | 0.010 | 0.950 | 0.005 |
| Accuracy | 0.905 | -0.018 | 0.979 | -0.002 | 0.951 | -0.015 |
| Balanced accuracy | 0.905 | 0.002 | 0.979 | 0.007 | 0.951 | 0.000 |
| AUC | 1.000 | 0.000 | 0.991 | 0.003 | 0.987 | -0.001 |

| Table 4.8 Feature Importance for Balanced Data with Outliers | | | |
| --- | --- | --- | --- |
|  | LR | DT | RF |
| 1 | Total Dialogue (60.24***) | Offline connection (1) | Offline connection (24%) |
| 2 | Patient posts (-49.59***) | Social return (2) | Response rate (21%) |
| 3 | Response rate (-4.90***) | Total dialogue (2) | Total Dialogue (19%) |
| 4 | Offline connection (-3.91***) | Private (3) | PriorExam (14%) |
| 5 | Social return (-1.80***) | Response rate (3) | Patient posts (11%) |
| 6 | PriorExam (1.40***) | PriorExam (4) | Social return (6%) |
| 7 | Answer_frq (-0.67***) | Question_frq (5) | Question_frq |
| 8 | Private (-0.61***) | Answer_frq (8) | Private |
| 9 | Question_frq (-0.54***) | Title 1 (8) | Answer_frq |
| 10 | Ranking2 (-0.23***) | Patient posts (9) | Title1 |
| 11 | Title 1 (-0.11***) | Ranking 2 (9) | Ranking2 |

*Note.* LR-logistic regression. Coefficients are shown in the brackets. DT – (simple) decision tree. The numbers in the brackets show the highest level of tree splits; RF – random forest. The numbers in the brackets show the percentage feature importance (only those above 5% are shown).
